# Predictors of Suicidal Thoughts and Attempts among School-Going Thai Adolescents: A Sex-Specific Structural Equation Modelling Analysis

**DOI:** 10.1007/s10578-024-01790-3

**Published:** 2024-11-23

**Authors:** Omid Dadras

**Affiliations:** 1 Department of Global Public Health and Primary Care, University of Bergen, Årstadveien 17, 5009 Bergen, Norway; 2 Research Center for Child Psychiatry, University of Turku, Turku, Finland

**Keywords:** Suicidal behaviors, Adolescents, Thailand, Predictors

## Abstract

Adolescent suicide is a growing public health concern in Thailand. Identifying predictors of suicidal behaviors is essential for targeted prevention strategies. Using Structural Equation Modeling, this study explores the predictors of suicidal thoughts and attempts among a sample of 5657 Thai adolescents in grades 7–12, typically representing the age group 13–17 years, with nearly equal proportions of males (47%) and females (53%) participants. Data from the Thailand Global School-based Student Health Survey (GSHS) 2021 were analyzed. Explanatory Factor Analysis (EFA) identified key constructs, followed by SEM stratified by sex to examine relationships between constructs and suicidal behaviors. Reliability and validity were assessed using Cronbach’s α and SEM fit indices. EFA identified five constructs: current substance use, history of violence, experiences of bullying, parental awareness, and poor mental health. SEM revealed poor mental health as a significant predictor for both sexes. Substance use, experiences of bullying, and a history of violence predicted suicidal behaviors in females. Parental vigilance was only a protective factor for males. The findings underscore the importance of gender-specific interventions and comprehensive mental health support to address the unique vulnerabilities of Thai adolescents, reducing suicidal thoughts and attempts.

**Supplementary Information:**

The online version contains supplementary material available at 10.1007/s10578-024-01790-3.

## Introduction

Adolescence is a critical period marked by significant physical, cognitive, and emotional changes [10]. These transitions can make individuals vulnerable to mental health issues, including depression, anxiety, and suicidal ideation [27]. Suicide is one of the leading causes of death among adolescents, highlighting the urgency of this public health concern [44]. The key risk factors for adolescent suicide encompass a range of mental health disorders, previous suicidal behaviors, adverse experiences, social isolation, access to lethal means, and other factors. Mental health issues like depression, anxiety, bipolar disorder, and substance abuse disorders significantly increase the risk of suicidal behavior [18]. Adverse experiences such as abuse, neglect, bullying, family conflicts, and exposure to intimate partner violence or sexual abuse are associated with increased suicidal thoughts and attempts [31]. Social isolation, lack of supportive relationships, and barriers to accessing mental health care contribute to suicidal behavior [31]. Understanding the factors that contribute to suicidal thoughts and attempts during adolescence is crucial for developing effective prevention and intervention strategies.

Thailand is grappling with alarmingly high suicide rates, which have been steadily increasing in recent years, particularly since the COVID-19 pandemic [42]. In 2022, Thailand's suicide rate reached 7.97 per 100,000 people, a concerning rise from 6.64 in 2019 [42]. The World Health Organization (WHO) estimates Thailand's suicide rate to be even higher at 8.8 per 100,000 people in 2019, potentially due to underreporting or misclassification [5]. Men in Thailand have a significantly higher suicide rate of 12.27 per 100,000 compared to 2.68 for women [5]. Adolescents and school-age children are among the most vulnerable groups for suicide attempts in Thailand [42]. According to the 2021 Global School-based Student Health Survey, 11% of males and 23% of females aged 13–17 considered suicide, with 12% of males and 18% of females attempting it. These concerning rates highlight the urgent need for further investigation into the underlying risk factors. Several unique cultural, social, and economic factors make Thailand particularly vulnerable to these trends. Strong collectivist values and the emphasis on family reputation can place intense pressure on adolescents to conform to societal expectations, leading to feelings of shame and hopelessness when these expectations are not met [9, 33]. Additionally, mental health stigma and traditional family structures often limit open communication about personal struggles, making it difficult for adolescents to seek help [23]. Economic pressures, heightened by the COVID-19 pandemic, have exacerbated stress and anxiety, further contributing to mental health issues [7]. Moreover, the rise of cyberbullying and exposure to violence in Thailand's rapidly digitalizing society presents unique challenges [33]. These factors, combined with limited access to mental health resources, particularly in rural areas [23], underscore the urgent need for targeted prevention and intervention strategies to address the growing issue of adolescent suicidality in Thailand.

Recent research highlights the importance of distinguishing between suicide ideators and attempters to better understand and prevent suicidal behavior. The ideation-to-action framework posits that the development of suicidal thoughts and the progression to attempts are distinct processes with different predictors, each influenced by different predictors [21, 22]. Suicidal thoughts, or ideation, refer to the contemplation of suicide, while suicide attempts involve actual efforts to end one's life. Previous studies have shown that mental health disorders, experiences of bullying, and substance use can be significant predictors of suicidal ideation and attempts. However, the distinction and nature of these relationships can vary based on factors such as gender and other demographic characteristics [15, 20], highlighting the need for a nuanced approach to research and intervention.

This distinction is particularly relevant in the context of Thailand, where adolescents face unique challenges, including academic pressure, social isolation, and limited access to mental health resources [6, 43]. Although previous literature highlights various factors associated with suicidal behaviors among adolescents [18, 30, 36, 41], much of this research has employed traditional statistical methods, such as logistic regression, to identify predictors of suicidal thoughts and attempts. While regression analysis provides valuable insights, it is often limited in its ability to uncover the complex, multidimensional relationships among variables. Approaches like structural equation modeling can help identify underlying latent constructs and unobserved heterogeneity in the data, which may be crucial for understanding the multidimensional nature of adolescent suicidality [12]. Therefore, in this study, we employ Structural Equation Modeling (SEM) to explore the constructs − poor mental health, substance use, experiences of bullying, and history of violence − that are represented by variables hypothesized to be associated with suicidal thoughts and attempts among school-going adolescents in Thailand. SEM allows for assessing two key components. First, the measurement model verifies how well the observed variables represent the underlying latent constructs (e.g., psychological traits or behaviors that are not directly measurable). Second, the structural model tests the relationships between these latent constructs and suicidal behaviors, accounting for measurement error and the interdependencies among the predictors, offering a more accurate understanding of these relationships. Our study aims to provide a deeper understanding of the factors contributing to suicidal thoughts and attempts among Thai adolescents. This research not only contributes to the existing body of knowledge but also offers practical implications for developing targeted interventions to reduce the suicide burden in this vulnerable population.

## Methods

### Data Source

We used the data from the Thailand Global School-based Student Health Survey (GSHS) 2021, a nationally representative survey conducted among school-based participants in grades 7–12, typically targeting adolescents aged 13–17 years. The survey covered various areas, including alcohol use, dietary habits, drug use, hygiene, mental health, physical activity, protective factors, sexual behaviors, tobacco use, and experiences of violence and unintentional injury. After obtaining informed consent, students were approached in the classroom setting, where they were provided with the questionnaire and recorded their responses on a computer-scannable answer sheet.

### Sampling and Response Rate

The GSHS 2021 employed a two-stage cluster sampling approach to ensure comprehensive representation of students in grades 7–12 across Thailand. Initially, schools were selected based on their enrollment size using probability proportional to size sampling. Subsequently, classes within the selected schools were randomly chosen, and all students within those classes were eligible to participate. The survey achieved an impressive school response rate of 92% and a student response rate of 90%, resulting in an overall response rate of 83%. A total of 5,657 students participated in the Thailand GSHS 2021 survey.

### Study Variables

The outcome variables included suicide thoughts and attempts. Suicide thoughts were measured by asking the question “During the past 12 months, did you ever seriously consider attempting suicide?” and responses were coded as 0 “no” and 1 “yes”. The suicide attempt was measured by asking the question “During the past 12 months, how many times did you actually attempt suicide?” with alternative responses no coded as “0” and yes coded as “1”. The independent variables were selected based on a comprehensive literature review, availability of variables in the Thailand GSHS 2021 survey, and theoretical relevance with latent construct and were recoded as binary as depicted in Table 1.

**Table 1:** Domains (latent variables) and items (observed variables) related to suicidal thoughts among school-going adolescents, Thailand GSHS 2021

Latent variables	Items (label)	Question	Recode
Poor mental health	Depression (depress)	During the past 12 months, how often have you felt lonely?	1 “most of the time/always”, 0 “never/rarely”
	Anxiety-induced insomnia (anxiety)	During the past 12 months, how often have you been so worried about something that you could not sleep at night?	1 “most of the time/always”, 0 “never/rarely”
Parental vigilance	Parental supervision (pcheck)	During the past 30 days, how often did your parents or guardians check to see if your homework was done?	1 “most of the time/always”, 0 “never/rarely”
	Parental understanding (punders)	During the past 30 days, how often did your parents or guardians understand your problems and worries?	1 “most of the time/always”, 0 “never/rarely”
	Parental awareness (pknow)	During the past 30 days, how often did your parents or guardians really know what you were doing with your free time?	1 “most of the time/always”, 0 “never/rarely”
Experiences of Bullying	Bullied at school (binscho)	During the past 12 months, have you ever been bullied on school property?	1 “yes”, 0 “no”
	Bullied outside school (boutscho)	During the past 12 months, have you ever been bullied when you were not on school property?	1 “yes”, 0 “no”
	Cyberbullied (cyberb)	During the past 12 months, have you ever been cyber bullied?	1 “yes”, 0 “no”
Current substance use	Cigarette smoking (cigar)	During the past 30 days, on how many days did you smoke cigarettes?	1 “at least one day”, 0 “0 days”
	Other tobacco products use (othertob)	During the past 30 days, on how many days did you use any tobacco products other than cigarettes, such as roll-your-own tobacco or baraku?	1 “at least one day”, 0 “0 days”
	Marijuana use (marijuana)	During the past 30 days, how many times have you used marijuana (also called weed)?	1 “at least one time”, 0 “0 time”
	Current alcohol use (alcohol)	During the past 30 days, on how many days did you have at least one drink containing alcohol?	1 “at least one day”, 0 “0 days”
History of violence	being physically attacked (pattack)	During the past 12 months, how many times were you physically attacked?	1 “at least one time”, 0 “0 time”
	physical fight (pfight)	During the past 12 months, how many times were you in a physical fight?	1 “at least one time”, 0 “0 time”
	Seriously injured (sinjured)	During the past 12 months, how many times were you seriously injured?	1 “at least one time”, 0 “0 time”
–	Missed school (qn53)*	During the past 30 days, on how many days did you miss classes or school without permission?	1 “at least one day”, 0 “0 day”
–	No close friend (qn27)*	How many close friends do you have?	1 “yes”, 0 “no”

^*^ These variables were excluded either due to the loading factor < 0.4 or loading on more than one factor

### Statistical Analysis

**Fig. 1:**
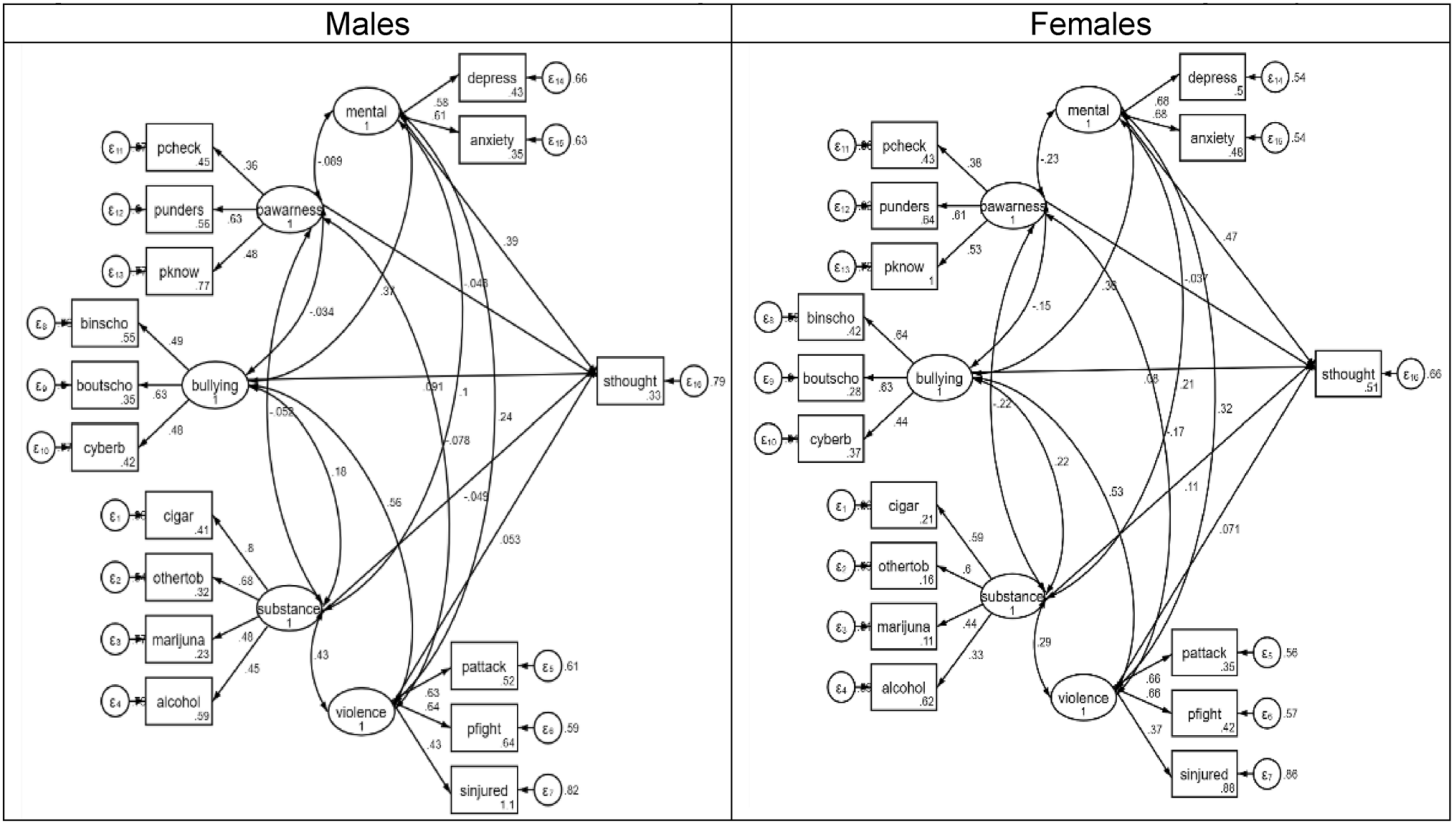
Structural equation models (SEM) diagrams for predictors of suicidal thoughts; by sex

**Fig. 2:**
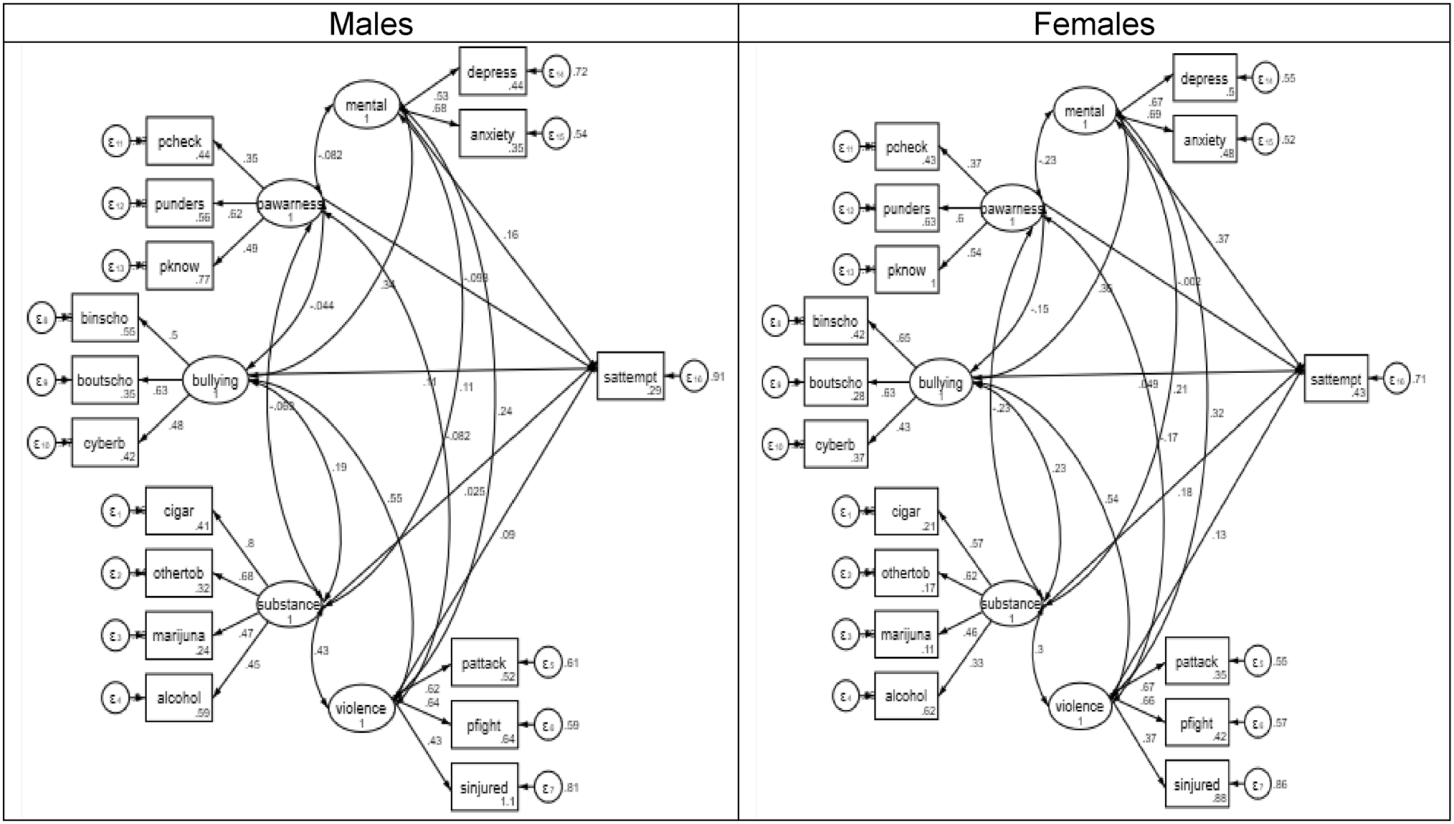
Structural equation models (SEM) diagrams for predictors of suicidal attempts; by sex

Descriptive statistics were utilized to characterize the participants and are presented in Table 1. Following a comprehensive literature review, relevant variables associated with suicidal behaviors were extracted from the Thailand GSHS 2021 dataset and prepared for factor analysis (Table 1). A correlation matrix of the observed variables used in the Exploratory Factor Analysis (EFA) was generated, showing moderate to strong correlations, supporting factor analysis. The matrix is provided in Supplementary Table S1. Exploratory Factor Analysis (EFA), employing the principal component factor method with oblique rotation, was conducted to uncover the underlying constructs. Dichotomizing items, consistent with the original GSHS response format, simplified the interpretation and facilitated the analysis by creating binary variables that align with the nature of the constructs being studied, such as the presence or absence of suicidal thoughts. The suitability of the data for EFA was verified using the Kaiser–Meyer–Olkin (KMO) test of sampling adequacy (KMO = 0.73, where values greater than 0.70 indicate suitability) and Bartlett's test of sphericity (p < 0.001, with p-values < 0.05 considered favorable) [14]. To enhance the interpretability of the factors, oblique rotation was applied to account for potential correlations between factors. Factors were retained based on eigenvalues (> 1), scree plot, and the amount of explained variance by each factor. Two variables (missed school and no close friend) were excluded either due to the loading factor < 0.4 or loading on more than one factor as recommended by previous literature [40]. Internal reliability was assessed using Cronbach’s α test (α = 0.77, indicating acceptable reliability). The construct validity of the factors emerging from EFA was examined using Structural Equation Modeling (SEM) analysis, stratified by sex. For each sex, two models were specified: one for suicidal thoughts and one for suicidal attempts, based on the ideation-to-action framework, which suggests that the development of suicidal ideation and the transition from ideation to attempts are distinct processes with potentially different predictors that may vary by sex [20, 35]. The SEM models included five latent variables/constructs identified from the EFA results: current substance use, history of violence, experiences of bullying, parental awareness, and poor mental health. These latent variables were operationalized through their respective observed variables, indicating that changes in the latent constructs are expected to lead to changes in the observed indicators. Model fit was assessed using the Root Mean Squared Error of Approximation (RMSEA) Standardized Root Mean Squared Residual (SRMR), the Coefficient of Determination (CD), Comparative Fit Index (CFI), Tucker-Lewis index (TLI); with an RMSEA < 0.05, SRMR < 0.08, a CD value close to 1, and CFI and TLI > 0.90 indicating a good fit. Additionally, for each model, R^2^ values were calculated to assess the proportion of variance explained by the latent constructs in predicting suicidal thoughts and suicidal attempts, serving as a measure of model fit. Diagrams illustrating the relationships between observed and latent variables, as well as the covariate structures, are presented in Figs. 1, 2. All analyses were performed in STATA 18 (College Station, TX: StataCorp LLC) and the p-value < 0.05 was considered statistically significant (Table 2).

**Table 2:** Sample characteristics, Thailand GSHS 2021

	N (%)
Age group	
< 14	1671 (22.9)
14–15	2182 (41.3)
≥ 16	1804 (35.8)
Sex	
Male	2504 (46.8)
Female	3135 (53.1)
Grade	
7th	1743 (22.5)
8th	905 (21.8)
9th	1288 (21.1)
10th	573 (12.3)
11th	509 (11.4)
12th	637 (10.8)
Total	5657 (100)

NB. The sum of numbers may not add up to the total number due to missing observations

## Results

### Sample Characteristics

The sample consisted of 5657 students, with a slightly higher proportion of females (53.1%) compared to males (46.8%). The majority of participants were aged 14–15 years (41.3%), followed by those aged ≥ 16 years (35.8%) and < 14 years (22.9%). Students were distributed across grades 7 to 12, with the largest group being in grade 7 (22.5%).

### Predictors of Suicidal Thoughts

As presented in Table 3, among male students, current substance use (β = -0.05, p = 0.177), parental vigilance (β = -0.05, p = 0.261), experiences of bullying (β = 0.09, p = 0.289), and history of violence (β = 0.05, p = 0.468) were not associated with suicidal thoughts. On the other hand, poor mental health was significantly associated with suicidal thoughts (β = 0.39, p < 0.001).

**Table 3:** Structural Equation Model (SEM) results for predictors of suicidal thoughts in Thai adolescents; by sex

	Males		Females	
Variable	Coeff. (95% CI) ^a^	p-value	Coeff. (95% CI) ^a^	p-value
cigar—> substance	0.80 (0.72, 88)	< .001	0.59 (0.42, 0.76)	< .001
othertob—> substance	0.68 (0.59, 0.77)	< .001	0.60 (0.48, 0.72)	< .001
marijuana—> substance	0.48 (0.31, 0.65)	< .001	0.44 (0.28, 0.60)	< .001
alcohol—> substance	0.45 (0.35, 0.56)	< .001	0.33 (0.26, 0.41)	< .001
substance—> sthought	−0.05 (-0.12, 0.02)	0.177	0.11 (0.03, 0.20)	0.012
pattack—> violence	0.63 (0.58, 0.68)	< .001	0.66 (0.59, 0.73)	< .001
pfight—> violence	0.64 (0.56, 0.71)	< .001	0.66 (0.58, 0.74)	< .001
sinjured—> violence	0.43 (0.35, 0.51)	< .001	0.37 (0.32, 0.42)	< .001
violence—> sthought	0.05 (-0.09, 0.20)	< .468	0.07 (-0.01, 0.16)	0.065
binscho—> bullying	0.49 (0.39, 0.58)	< .001	0.64 (0.55, 0.74)	< .001
boutscho—> bullying	0.63 (0.55, 0.72)	< .001	0.63 (0.58, 0.69)	< .001
cyberb—> bullying	0.48 (0.37, 0.59)	< .001	0.44 (0.36, 0.51)	< .001
bullying—> sthought	0.09 (-0.08, 0.26)	0.289	0.08 (0.002, 0.16)	0.046
pcheck—> pawarness	0.36 (0.27, 0.44)	< .001	0.38 (0.31, 0.45)	< .001
punders—> pawarness	0.63 (0.46, 0.80)	< .001	0.61 (0.46, 0.75)	< .001
pknow—> pawarness	0.48 (0.33, 0.64)	< .001	0.53 (0.39, 0.66)	< .001
pawarness—> sthought	−0.05 (-0.13, 0.04)	0.261	-0.04 (-0.09, 0.2)	0.175
depress—> mental	0.58 (0.46, 0.71)	< .001	0.68 (0.63, 0.73)	< .001
anxiety—> mental	0.61 (0.47, 0.74)	< .001	0.68 (0.62, 0.74)	< .001
mental—> sthought	0.39 (0.29, 0.49)	< .001	0.47 (0.40, 0.53)	< .001
Goodness-of-Fit				
SRMR ^b^	0.033		0.033	
CD ^c^	0.989		0.989	

^a^ Standardized coefficient (95% confidence interval). ^b^ SRMS: Standardized root mean squared residual which measures the average standardized residual. Values < 0.08 indicate a good fit. ^c^ *CD* coefficient of determination reflects the proportion of variance explained by the model and values close to 1 indicate a high level of explained variance

Among female students, current substance use (β = 0.11, p = 0.012), experiences of bullying (β = 0.08, p = 0.046), and poor mental health (β = 0.47, p < 0.001) were associated with suicidal thoughts. However, a history of violence (β = 0.07, p = 0.065) and parental vigilance (β = − 0.04, p = 0.175) were not significant.

### Predictors of Suicidal Attempts

As presented in Table 4, among male students, current substance use (β = 0.02, p = 0.501), experiences of bullying (β = 0.11, p = 0.209), and history of violence (β = 0.09, p = 0.156) were not associated with suicidal attempts. Poor mental health (β = 0.16, p < 0.012) was associated with suicidal attempts, while parental vigilance (β = −0.09, p = 0.007) was protective against it.

**Table 4:** Structural Equation Model (SEM) results for predictors of suicidal attempts in Thai adolescents; by sex

	Males		Females	
Variable	Coeff. (95% CI) ^a^	p-value	Coeff. (95% CI) ^a^	p-value
cigar—> substance	0.80 (0.72, 88)	< .001	0.57 (0.39, 0.74)	< .001
othertob—> substance	0.68 (0.60, 0.76)	< .001	0.62 (0.51, 0.74)	< .001
marijuana—> substance	0.47 (0.31, 0.64)	< .001	0.46 (0.31, 0.60)	< .001
alcohol—> substance	0.45 (0.35, 0.55)	< .001	0.33 (0.26, 0.40)	< .001
substance—> sthought	0.02 (-0.05, 0.01)	0.501	0.18 (0.04, 0.32)	0.013
pattack—> violence	0.62 (0.57, 0.68)	< .001	0.67 (0.60, 0.74)	< .001
pfight—> violence	0.64 (0.57, 0.72)	< .001	0.66 (0.58, 0.74)	< .001
sinjured—> violence	0.43 (0.36, 0.51)	< .001	0.37 (0.33, 0.42)	< .001
violence—> sthought	0.09 (-0.04, 0.22)	< .156	0.13 (0.05, 0.22)	0.003
binscho—> bullying	0.50 (0.41, 0.59)	< .001	0.64 (0.56, 0.73)	< .001
boutscho—> bullying	0.63 (0.54, 0.73)	< .001	0.63 (0.58, 0.69)	< .001
cyberb—> bullying	0.48 (0.37, 0.59)	< .001	0.43 (0.35, 0.51)	< .001
bullying—> sthought	0.11 (-0.06, 0.28)	0.209	0.05 (-0.07, 0.16)	0.388
pcheck—> pawarness	0.35 (0.27, 0.44)	< .001	0.37 (0.30, 0.44)	< .001
punders—> pawarness	0.62 (0.47, 0.77)	< .001	0.60 (0.46, 0.74)	< .001
pknow—> pawarness	0.49 (0.34, 0.64)	< .001	0.54 (0.39, 0.68)	< .001
pawarness—> sthought	-0.09 (-0.16, -0.03)	0.007	-0.002 (-0.05, 0.04)	0.934
depress—> mental	0.53 (0.37, 0.69)	< .001	0.67 (0.61, 0.73)	< .001
anxiety—> mental	0.68 (0.46, 0.90)	< .001	0.69 (0.62, 0.76)	< .001
mental—> sthought	0.16 (0.04, 0.29)	< .012	0.37 (0.31, 0.44)	< .001
Goodness-of-Fit				
SRMR ^b^	0.034		0.033	
CD ^c^	0.989		0.989	

^a^Standardized coefficient (95% confidence interval). ^b^ SRMS: Standardized root mean squared residual which measures the average standardized residual. Values < 0.08 indicate a good fit. ^c^ CD: Coefficient of Determination reflects the proportion of variance explained by the model and values close to 1 indicate a high level of explained variance

Among female students, current substance use (β = 0.18, p = 0.013), history of violence (β = 0.13, p = 0.003) and poor mental health (β = 0.37, p < 0.001) were significantly associated with suicidal attempts. However, experiences of bullying (β = 0.05, p = 0.388), and parental vigilance (β = − 0.002, p = 0.934) were not significant.

## Discussion

This study makes significant contributions to the existing literature on adolescent suicidality by providing gender-specific insights that can inform targeted prevention and intervention strategies. The findings reveal that while poor mental health is a strong predictor of suicidal thoughts and behaviors for both male and female adolescents, other risk factors, such as substance use and experiences of bullying, play a more prominent role in predicting suicidality among females. In contrast, parental vigilance is found to be a protective factor only among male adolescents. These gender-specific differences underscore the necessity of designing suicide prevention programs that are tailored to address the unique needs of each gender.

For both male and female adolescents, poor mental health emerged as a significant predictor of suicidal thoughts and attempts. This aligns with previous studies underscoring the critical role of mental health issues, such as depression and anxiety, in increasing suicide risk among adolescents [30, 35, 36, 41]. Mental health issues such as depression and anxiety can negatively impact coping mechanisms. Teenagers struggling with mental health problems may lack healthy coping skills to manage stress, overwhelming emotions, or difficult life experiences, making suicide seem like an escape, and increasing the risk of suicidal thoughts and behaviors [11, 25]. The dynamics of suicidal attempts can; however, differ significantly between males and females, influenced by various social, psychological, and biological factors [34]. For example, males may feel pressured to conform to traditional notions of masculinity, which discourage emotional expression and vulnerability, potentially leading to a higher risk of impulsive suicidal attempts when faced with crises. Females, on the other hand, may experience greater social support networks but are also more susceptible to relational issues, such as bullying or family conflict as observed in our study, which can exacerbate feelings of hopelessness and despair and consequent suicidal behaviors [37].

Unlike male students in this study, among female students, current substance use was significantly associated with both suicidal thoughts and attempts. The relationship between substance use and suicidal behavior is a complex and multifaceted issue, and it is important to consider the role of gender in this dynamic. Research has shown that among females, current substance use is significantly associated with both suicidal thoughts and attempts, a pattern that is not as pronounced among males [4, 39]. This may suggest that the impact of substance use on suicidal behavior may be more pronounced in female students compared to their male counterparts. One potential explanation for this gender disparity is the complex interplay between biological, psychological, and social factors that contribute to substance use disorders and suicidal behavior. For instance, women may be more susceptible to the negative emotional effects of substance use, which could increase their risk of suicidal ideation and attempts [4, 19], R. K. [26]. Additionally, females may be more likely to use substances as a means of self-medicating mood disturbances, increasing their risk of suicidal behavior [8]. Gender norms and societal expectations may shape how men and women cope with substance use and mental health issues, with women potentially more inclined to engage in self-destructive behaviors (R. K. [26]). Evidence suggests that women often experience more severe medical, psychiatric, and functional consequences associated with substance use disorders, which could contribute to the heightened risk of suicidal behavior [19], R. Kathryn [26]. This suggests a need for gender-specific prevention strategies such as tailored interventions addressing mental health and social pressures, substance use education highlighting its risks, and early screening and support among female students with substance use issues.

Experiences of bullying and a history of violence were associated with suicidal thoughts among female students but not male students. These differences indicate that females may be more sensitive to relational and social stressors, whereas males might respond differently to these experiences [17]. Females may be more susceptible to relational aggression (bullying that involves harming social relationships) and social exclusion tactics often used in bullying [2, 17]. This could heighten feelings of isolation and despair, increasing suicide risk. Males might respond to bullying and violence more outwardly, while females may internalize these experiences, leading to depression and suicidal ideation [1, 2]. Implementing gender-sensitive bullying prevention programs, promoting social-emotional learning and resilience-building initiatives, and mandating trauma-informed approaches in schools to address the unique vulnerabilities of female students to relational aggression, bullying, and associated suicidal ideation [19, 38]. Additionally, ensuring access to comprehensive mental health screening, support services, and staff training to identify and intervene in cases of bullying, violence, and suicidal thoughts among female students [13, 38].

Parental vigilance was found to be protective against suicidal attempts among male students but not female students. This suggests that parental involvement and monitoring may be more effective in mitigating suicide risk among males, highlighting the importance of family-based interventions [32]. Previous studies found that females may respond better to open communication about emotions, while males might benefit more from indirect approaches like monitoring activities [28, 32]. Parental monitoring might encourage help-seeking behaviors in boys who are typically less likely to express emotional distress [3, 28, 32]. However, it is important to consider that parental vigilance can also have potential negative implications. For some adolescents, excessive monitoring or intrusive supervision might lead to feelings of distrust or invasion of privacy, which could exacerbate feelings of isolation or resentment [24]. Such negative effects could counteract the protective benefits and possibly contribute to adverse outcomes. This is particularly relevant for females, who may respond better to open communication about emotions rather than strict monitoring [16]. These findings suggests traditional approaches to parental involvement might not be equally effective for male and female adolescents. Therefore, gender-tailored strategies such as designing workshops and programs that teach parents how to monitor and communicate with their sons and daughters in ways that resonate with each gender [29]. Collaboration between schools, mental health services, and community organizations is crucial to provide families with the knowledge and resources they need to address the unique needs of their children.

### Limitation and Strengths

Some limitations must be considered in the interpretation of results. First, the reliance on self-reported data for mental health issues, substance use, and suicidal behaviors may introduce response biases such as social desirability bias or recall bias. Adolescents might underreport or overreport their behaviors and experiences due to stigma or memory lapses. Second, the study is based on data from the Global School-based Student Health Survey (GSHS) 2021, which targets school-going adolescents. Therefore, the findings may not be generalizable to out-of-school adolescents or those in different educational settings in Thailand. Third, some important variables that could influence suicidal behavior, such as family dynamics, socio-economic status, and access to mental health services, were not included in the GSHS survey. Additionally, the measurement of suicidal thoughts and attempts relied on binary yes/no questions from the GSHS, rather than utilizing more detailed and validated scales like the Modified Scale for Suicide Ideation (MSSI). These limitations are inherent to the secondary data source used and may affect the depth of insights gained from the analysis and may limit the comprehensiveness of the analysis. Fourth, findings are specific to Thai adolescents and may not be directly applicable to adolescents in other cultural contexts due to differences in societal norms, mental health stigmas, and substance use patterns. Cross-cultural studies are needed to explore these aspects further. Fifth, although the study controls for several confounding factors, there may still be unmeasured variables that influence the observed relationships. For example, genetic predispositions, personality traits, and other socioenvironmental factors could play a role in suicidal behaviors. Lastly, This study highlights significant sex differences in predictors of suicidal behavior, however, it does not delve deeply into the underlying mechanisms driving these differences. Further research is needed to understand the complex interplay of biological, psychological, and social factors that contribute to these gender-specific patterns.

Despite these limitations, this study is the first study in Thailand that used SEM analysis, allowing for a comprehensive analysis of the complex, multidimensional relationships among variables related to suicidal thoughts and attempts among Thai adolescents. Additionally, the study's sex-specific approach provides valuable insights into the differing predictors of suicidal behavior for male and female students, emphasizing the necessity for gender-tailored interventions, and informing the development of targeted prevention and intervention strategies.

### Summary

This study aimed to identify the factors contributing to suicidal thoughts and attempts among school-going Thai adolescents. The findings underscore the significant role of poor mental health as a predictor of suicidal behaviors across both male and female adolescents. Notably, current substance use was identified as a significant predictor of suicidal thoughts and attempts among female students, but not males, suggesting a gender disparity that underscores the need for tailored interventions. Additionally, experiences of bullying and a history of violence were associated with suicidal thoughts among female students, pointing to their heightened sensitivity to relational and social stressors compared to their male counterparts. Parental vigilance emerged as a protective factor against suicidal attempts among male students, indicating the effectiveness of family-based interventions in mitigating suicide risk. Prevention efforts for female adolescents should include components that address substance use prevention and anti-bullying strategies, recognizing their heightened vulnerability to these factors. Meanwhile, interventions for male adolescents could focus on enhancing parental involvement and supervision, and promoting open communication between parents and sons to detect early signs of distress. Future research should continue to explore the underlying mechanisms driving these gender differences, considering cultural, social, and psychological factors that may influence how adolescents respond to stressors and protective factors. Additionally, there is a need for longitudinal studies to assess how these predictors of suicidality evolve over time, which could further inform the development of dynamic, adaptable intervention programs.

## Supplementary Information

Below is the link to the electronic supplementary material.

Supplementary file1 (RTF 115 KB)

## Data Availability

The GSHS 2021 is a publicly available dataset and is available on the World Health Organization NCD Microdata Repository website at: https://extranet.who.int/ncdsmicrodata/index.php/catalog/.
