# Supplementary material for: Predictors of Suicidal Thoughts and Attempts among School-Going Thai Adolescents: A Sex-Specific Structural Equation Modelling Analysis
Source: Child Psychiatry Hum Dev. 2024 Nov 23;57(4):1249–59. doi: 10.1007/s10578-024-01790-3 (PMC13437605; doi:10.1007/s10578-024-01790-3)
Supplement: Supplementary file 1 [file 10578_2024_1790_MOESM1_ESM.rtf]

Table S1. Correlation matrix of observed variables used in EFA
  Variables	 pattack	pfight	sinjured	cigar	othetob	marijuna	alcohol	pcheck	punders	pknow	binscho	boutscho	cyberb	depress	anxiety	
pattack	1.000	
pfight	0.439	1.000	
sinjured	0.267	0.274	1.000	
cigar	0.161	0.198	0.141	1.000	
othetob	0.133	0.203	0.150	0.531	1.000	
marijuna	0.082	0.100	0.123	0.264	0.160	1.000	
alcohol	0.118	0.165	0.087	0.323	0.317	0.201	1.000	
pcheck	0.001	-0.006	0.017	-0.033	-0.018	-0.053	0.002	1.000	
punders	-0.064	-0.082	-0.052	-0.039	-0.035	-0.056	-0.031	0.254	1.000	
pknow	-0.088	-0.084	-0.088	-0.109	-0.107	-0.084	-0.075	0.185	0.311	1.000	
binscho	0.228	0.221	0.139	0.052	0.055	0.022	0.011	-0.002	-0.042	-0.063	1.000	
boutscho	0.231	0.205	0.141	0.114	0.088	0.077	0.078	0.006	-0.036	-0.047	0.401	1.000	
cyberb	0.161	0.170	0.146	0.082	0.067	0.118	0.066	-0.024	-0.061	-0.043	0.223	0.274	1.000	
depress	0.110	0.088	0.094	0.026	0.017	0.059	0.010	-0.006	-0.083	-0.067	0.152	0.103	0.134	1.000	
anxiety	0.113	0.071	0.122	0.058	0.046	0.105	0.035	0.003	-0.060	-0.046	0.123	0.112	0.136	0.420	1.000
